# Transcriptome analysis reveals defense-related genes and pathways against *Xanthomonas campestris* pv. *vesicatoria* in pepper (*Capsicum annuum* L.)

**DOI:** 10.1371/journal.pone.0240279

**Published:** 2021-03-11

**Authors:** Shenghua Gao, Fei Wang, Juntawong Niran, Ning Li, Yanxu Yin, Chuying Yu, Chunhai Jiao, Minghua Yao

**Affiliations:** 1 Hubei Key Laboratory of Vegetable Germplasm Enhancement and Genetic Improvement, Cash Crops Research Institute, Hubei Academy of Agricultural Sciences, Wuhan, Hubei, China; 2 Kasetsart University, Bangkok, Thailand; Universidade de Lisboa Instituto Superior de Agronomia, PORTUGAL

## Abstract

Bacterial spot (BS), incited by *Xanthomonas campestris* pv. *vesicatoria* (*Xcv*), is one of the most serious diseases of pepper. For a comparative analysis of defense responses to *Xcv* infection, we performed a transcriptomic analysis of a susceptible cultivar, ECW, and a resistant cultivar, VI037601, using the HiSeq^TM^ 2500 sequencing platform. Approximately 120.23 G clean bases were generated from 18 libraries. From the libraries generated, a total of 38,269 expressed genes containing 11,714 novel genes and 11,232 differentially expressed genes (DEGs) were identified. Functional enrichment analysis revealed that the most noticeable pathways were plant-pathogen interaction, MAPK signaling pathway—plant, plant hormone signal transduction and secondary metabolisms. 1,599 potentially defense-related genes linked to pattern recognition receptors (PRRs), mitogen-activated protein kinase (MAPK), calcium signaling, and transcription factors may regulate pepper resistance to *Xcv*. Moreover, after *Xcv* inoculation, 364 DEGs differentially expressed only in VI037601 and 852 genes in both ECW and VI037601. Many of those genes were classified as NBS-LRR genes, oxidoreductase gene, WRKY and NAC transcription factors, and they were mainly involved in metabolic process, response to stimulus and biological regulation pathways. Quantitative RT-PCR of sixteen selected DEGs further validated the RNA-seq differential gene expression analysis. Our results will provide a valuable resource for understanding the molecular mechanisms of pepper resistance to *Xcv* infection and improving pepper resistance cultivars against *Xcv*.

## Introduction

Bell pepper (*Capsicum annuum* L.), an important member of the Solanaceae family, is one of the most important vegetable crops in China and many other countries [1]. It is rich in antioxidant compounds, such as capsanthin and capsaicin, which are essential for human health [1]. In the past few decades, many research efforts have been carried out to increase pepper production because of its high nutritional and commercial value. However, pepper production has not achieved its potential yield due to biotic stresses, like bacterial spot disease and anthracnose, and abiotic stresses like drought and salinity [2–4]. Thus, it is necessary to take more rigorous steps to improve the productivity of pepper.

Bacterial spot (BS), caused by gram-negative plant pathogenic bacterium *Xanthomonas campestris* pv. *vesicatoria* (*Xcv*), is a severe disease of pepper, resulting in the reduction in quality and quantity of the yield in many pepper production areas, especially during periods of high temperatures and high moisture [5, 6]. *Xcv* infection usually leads to dark lesions on the foliage and fruit of the plant. Besides, lesions coalescence and leaf death could occur in severe cases [7]. The occurrence of BS has been reported all over the world, such as the USA, north-western Nigeria and Saudi Arabia [8–11]. BS has also occurred in China and has become more and more serious in recent years, especially in southern China. The method for controlling BS relies upon an integrated approach, which includes intensive copper-based bactericidal application, crop rotation strategies, seed treatment and use of resistant cultivars [7]. However, the most cost-effective and environmentally sustainable solution is to use resistant varieties. Moreover, the copper-tolerance of *Xanthomonas* strains is continuously enhanced [7]. Thus, it is particularly necessary to breed disease-resistant varieties [11].

The development of BS resistant commercially valuable cultivars through molecular breeding has been going on for many years. Until now, five non-allelic dominant hypersensitive resistance genes, *Bs1*-*Bs4*, *Bs7* and two recessive non-hypersensitive resistance genes, *bs5* and *bs6*, have been used in pepper [7, 12, 13]. While *Bs2*, *Bs3*, *Bs4* and *Bs7* have been cloned, for their molecular markers for marker-assisted selection (MAS) were developed [5, 14–17]. Also, *bs5* has been mapped and its linked markers are available [18]. These five dominant loci (*Bs1*-*Bs4*, *Bs7*) have been shown to confer resistance to *Xcv* in a gene-for-gene manner [5, 7, 13–17, 19]. The executor R gene *Bs1* in the resistant genotype VI037601 of the pepper species *Capsicum annum* L. confers disease resistance to *Xcv* strain 23–1 harbouring the avirulence gene *avrBs1* [7]. Each of these single dominant genes described above individually confers resistance to several races of *Xcv*. However, each resistance gene can be overcome by specific races of the bacteria in field-grown plants [7]. A deeper understanding of the responses of plant hosts to bacterial infection in pepper will contribute to accelerate the molecular breeding process and to tackle the issue of the possible evolution of BS pathogens.

RNA-seq has been proven to be a robust and cost-effective tool for examining the quantity and sequences of RNA using next-generation sequencing (NGS), which has been widely used to study global expression profiles and reveal differentially expressed genes (DEGs) involved in resistance pathways under biotic and abiotic stress, such as in pepper [20–25]. In the case of BS stress mechanism, several transcriptomic studies have also been performed using microarrays and RNA-seq technique in tomato [26, 27].

However, a genome-wide and comprehensive analysis of genes respond to *Xcv* infection is not yet available in pepper. Therefore, in the present study, the transcriptome of two contrasting pepper genotypes (ECW and VI037601) inoculated with *Xcv* was sequenced using Illumina paired-end sequencing technology. We identified thousands of DEGs, which were evaluated by Gene Ontology (GO) and Kyoto Encyclopedia of Genes and Genomes (KEGG) enrichment analyses. Our findings could help explore the resistance-genes and biological pathways associated with the pepper bacterial spot disease, and in understanding the molecular mechanisms of pepper plants’ defenses against *Xcv*.

## Materials and methods

### Plant materials and pathogen inoculation

Two bell pepper genotypes, VI037601 and Early Calwonder (ECW), were used for transcriptomic analysis provided by World Vegetable Center, Thailand (https://avrdc.org/). Plants were grown under standard glasshouse conditions for 16 h lighting at 25°C/ 8 h darkness at 20°C in a relative humidity of approximately 60%. *Xcv* strain 23–1 was grown at 28°C on nutrient agar medium for two days, and then scraped into sterile water to make a suspension with a concentration of 2×10^8^ cfu/ml. The suspension was inoculated using a syringe on the abaxial leaf surface near the midrib of the third to fifth pepper leaves to form a 1.5-2cm diameter water-soaked area, when plants were at the five leaves stage. The leaf fragments within 2 cm of the *Xcv* infection site, were collected for RNA isolation at 0h, 6h and 24h post- inoculation (hpi), respectively. Pepper leaves of respective varieties at 0 hpi were used as control. Samples were collected from ECW and VI037601 leaves 0–2 cm away from the inoculation point at three different inoculation time points (0, 6, and 24 hours), which were named as ECW_0H, ECW_6H, ECW_24H, VI037601_0H, VI037601_6H, and VI037601_24H, respectively. The samples were immediately placed in liquid nitrogen and stored at -80˚C for RNA extraction and further analysis. Fifteen leaves randomly selected from five different plants were polled as a biological replicate. Three independent biological replicates were prepared for each treatment.

### RNA extraction, library construction and transcriptome sequencing

Total RNA was extracted from 18 leaf tissue samples, including 3 replicates of each treatment condition (3 time points × 2 genotypes), using the Trizol Reagent (Life Technologies, California, USA) according to the manufacturer’s instructions, and then treated with TURBO DNase I (Promega, Beijing, China) to remove genomic DNA contamination. The integrity and concentration of all RNA samples were examined by the 2100 Bioanalyzer (Agilent Technologies, Inc., Santa Clara, CA, USA) and 1.2% agarose gel electrophoresis. The prepared total RNA samples were sent to Frasergen Bioinformatics Co., Ltd (Wuhan, China) where the cDNA library was constructed using NEBNext® Ultra™ RNA Library Prep Kit for Illumina® (NEB, E7530) according to the manufacturer’s instructions. In brief, the first-strand and the second-strand cDNA were synthesized using approximately 250~300 bp RNA inserts, which were fragmented by the enriched mRNA. After end-repair/dA-tail and adaptor ligation, the suitable fragments of double-strand cDNA were isolated by Agencourt AMPure XP beads (Beckman Coulter, Inc.), and then enriched by PCR amplification. Finally, the purity and quality of the libraries were measured by Agilent 2100 Bioanalyzer and Qubit 2.0. The eighteen cDNA libraries prepared were sequenced by Biomarker Technologies (Wuhan, China) using the Illumina HiSeq 2500 platform with pair-end 150 nt. RNA-seq was performed as previously described [23]. The transcriptome sequencing data from this study were available from the NCBI SRA database under BioProject accession number PRJNA693027.

### Transcriptome analysis using reference genome-based reads mapping

The quality check was performed to eliminate low quality reads with the only adaptor, unknown nucleotides> 5%, or Q20< 20% using SOAPnuke-2.1.0 [28]. The high-quality clean reads that were filtered from the raw reads were mapped to the reference genome of cultivated pepper Zunla-1 (*C*. *annuum* L.) (https://www.pnas.org/content/111/14/5135) with TopHat 2.0 software [29–31]. Potential duplicate molecules were removed by examining aligned records from the aligners in BAM/SAM format [32]. Fragments per kilobase of transcript per million fragments mapped read (FPKM) values were used to calculate the gene expression levels based on Cufflinks software [33].

### Identification and functional analysis of DEGs

DEGs were identified using DEGseq2 in the four comparisons of ECW_6H-vs-ECW_0H, ECW_24H-vs-ECW_0H, VI037601_6H-vs-VI037601_0H and VI037601_24H-vs-VI037601_0H [34]. The fold change of genes was calculated based on the ratio of the FPKM values. The genes with an absolute value of |log2(fold change)|≥1 and the false discovery rate (FDR) values <0.05 were accepted to represent significant DEGs, which were used for further analysis.

To acknowledge the putative functions and pathways of the DEGs in above four comparisons, GO functional enrichment analysis was performed with Blast2GO (version 3.0) (https://www.blast2go.com/) [35]. KEGG pathways analysis of DEGs was carried out using Cytoscape software (version 3.2.0) (https://cytoscape.org/) with the ClueGO plugin by a hypergeometric test and the Benjamini-Hochberg FDR correction (FDR ≤ 0.05) [36].

### Identification of transcription factors (TFs)

Transcription factors were identified using PlantTFDB (http://planttfdb.gao-lab.org/), which included the sequences of 58 plant transcription factor families from 165 plant species [37]. The unigene sequence was compared with the transcription factor database by BLASTx alignment, and the gene with the best E-value less than 10^−5^ was selected as the annotation information of the unigenes.

### Quantitative RT-PCR (qRT-PCR) analysis

To validate the RNA-Seq data, the relative expression levels of randomly selected DEGs were examined by quantitative Real-time PCR (qRT-PCR). The corresponding mRNA sequences of the selected genes were searched from the Sol Genomics Network (SGN) (https://www.solgenomics.net/). All primers for qRT-PCR were designed according to the transcript sequences using Primer Premier 5.0 (http://www.premierbiosoft.com/). The primers used in this experiment are listed in S11 Table. Approximately 2 μg of total RNA was isolated from infected leaves of ECW and VI037601 by TRIzol reagent, which was used to synthesize the cDNA through the cDNA synthesis kit (TransGen, Beijing, China) according to the manufacturer’s instructions. Quantitative RT-PCR (qRT-PCR) was performed in 96-well plates on Thermo Fisher Scientific Biosystems QuantStudio 5 Real-Time PCR system (Applied Biosystem, MA, USA) using SYBR Premix Ex Taq™ Kit (Takara, Dalian, China). The protocols of qRT-PCRs were used as follows: 95°C for 5 min, followed by 40 cycles of 95°C for 10 s, 58°C for 20 s, and 72°C for 15 s, plus melting curves to verify PCR products. Ubiquitin-conjugating protein CaUbi3 (Accession Number: AY486137.1) was used as an internal reference [38]. Samples were collected as previous in this study, and three independent biological replicates were analyzed. The relative expression level of the selected genes was calculated with the 2^-ΔΔCT^ method [39].

## Results

### RNA sequencing of pepper leaves after Xcv infection and assembly of transcriptome

**Fig 1:**
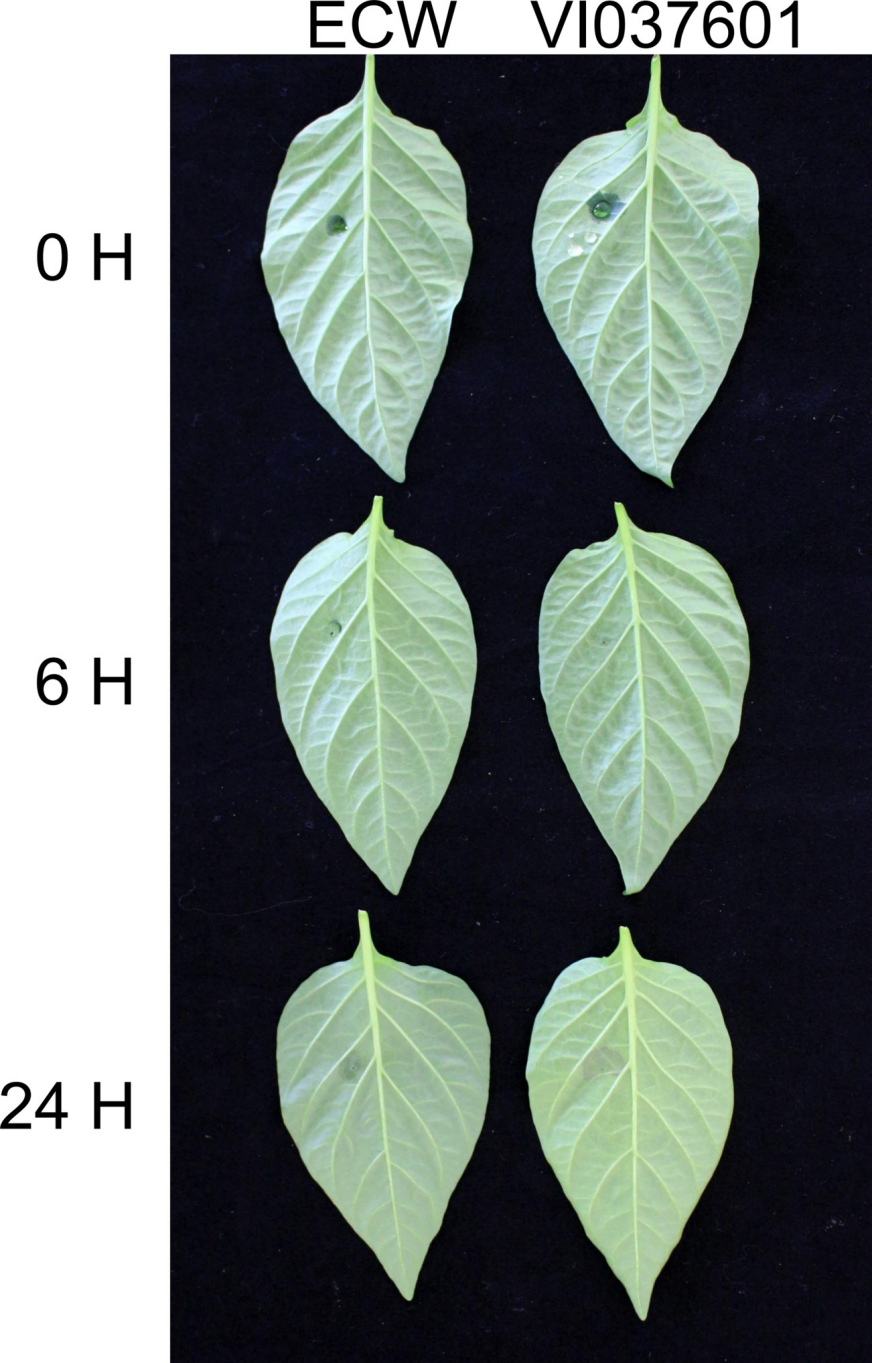
Reaction patterns of ECW and VI037601 to *Xcv* strain 23–1. 0, 6 hpi and 24 hpi represented 0 hour, 6 hours and 24 hours post *Xcv* inoculation with a needleless syringe, respectively.

First, to confirm the resistance of ECW and VI037601 to *Xcv*, an injection inoculation was carried out. The results showed that the symptoms were similar between resistant and susceptible plants by 24 hours post inoculation (hpi) (Fig 1). However, a hypersensitive response (HR) symptom was observed in VI037601 containing the R gene *Bs1* at 24 hpi, whereas cultivar ECW presented no HR symptom at either timepoints, indicating that VI037601 and ECW may have different responses to *Xcv* infection at the transcriptome level (Fig 1).

To accurately evaluate the comparative expression of genes in ECW and VI037601, eighteen cDNA libraries were used for RNA sequencing. Approximately 120.23 G clean bases were generated using an Illumina HiSeq 2500 sequencing platform. After quality control, each library contained between 22,079,427 to 27,158,399 clean read pairs. GC contents were ranged from 43.5% to 44.0% (S1 Table). The number of clean reads, 91%-94% were mapped to the pepper reference genome Zunla-1 (S1 Table). A total of 38,269 expressed genes, including 11,714 novel genes, were identified in this study (S2 and S3 Tables). There were 34,459, 34,699, 34,541, 34,727, 35,074, and 34,947 expressed genes in ECW_0H, ECW_6H, ECW_24H, VI03760_0H, VI03760_6H, and VI03760_24H, respectively (S3 Table).

### Expression analysis and identification of differentially expressed genes

**Fig 2:**
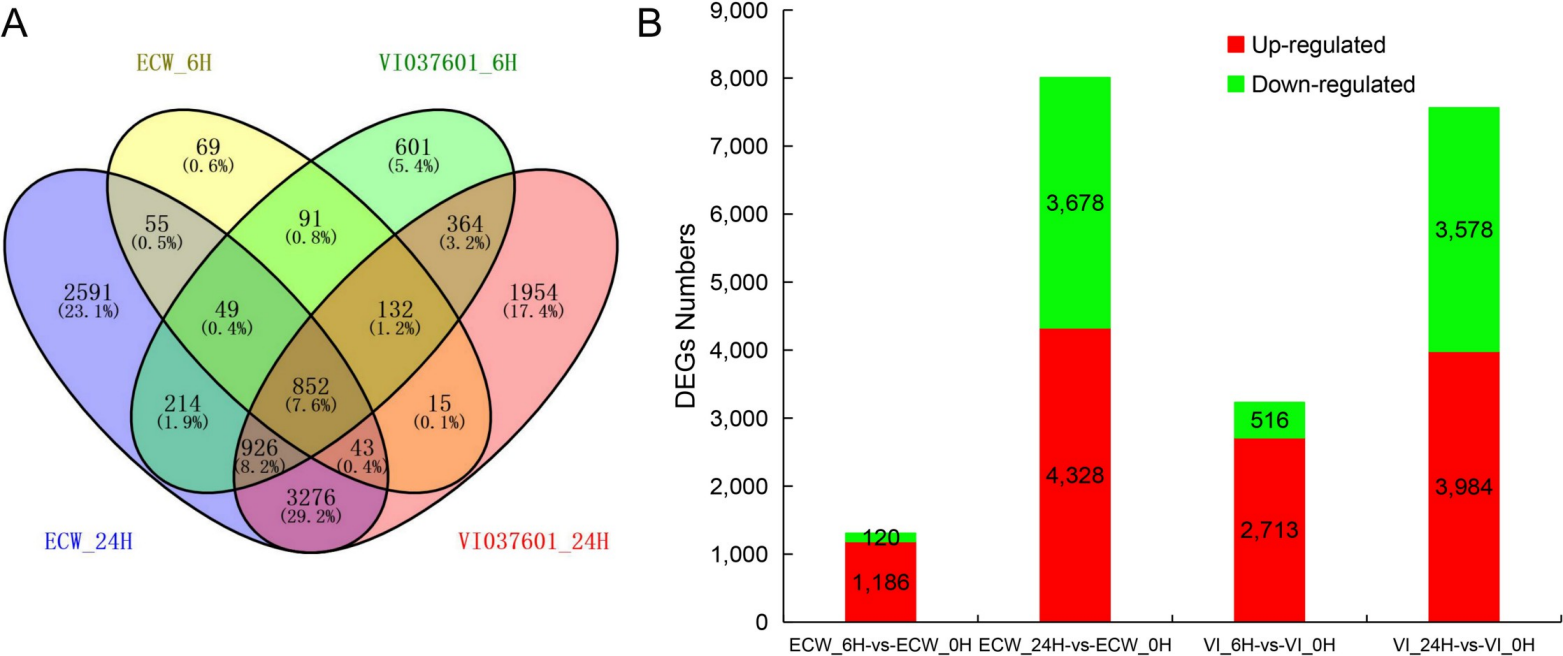
Expressional analysis of DEGs in ECW and VI037601 leaves at 6 hours and 24 hours post *Xcv* inoculation with *Xcv*. (**A**) Numbers of DEGs at 6 hpi and 24 hpi in ECW and VI037601, or between ECW and VI037601 at different time points. (**B**) Numbers of up- and down- regulated DEGs at 6 hpi and 24 hpi in ECW and VI037601, respectively.

To investigate the expression patterns of genes in pepper leaves during the different stages after *Xcv* infection, a total of 11,232 DEGs were identified in ECW and VI037601 at 6 hpi and 24 hpi, including 3,361 novel differentially expressed genes (S4 Table). Among them, 1,306 (1,186 up regulated and 120 down regulated) and 8,006 (4,328 up regulated and 3,678 down regulated) DEGs were found at 6 hpi and 24 hpi in ECW, respectively. 3,229 (2,713 up regulated and 516 down regulated) and 7,562 (3,984 up regulated and 3,578 down regulated) DEGs were identified at 6 hpi and 24 hpi in VI037601, respectively (Fig 2A and 2B and S4 Table). However, 999 (920 commonly up regulated and 73 commonly down regulated) and 2,274 (1,953 commonly up regulated and 290 commonly down regulated) DEGs overlapped at 6 hpi and 24 hpi in ECW and VI037601, respectively (Fig 2A and S4 Table). Interestingly, 852 overlapping DEGs were found in ECW and VI037601 post *Xcv* inoculation (Fig 2A and S5 Table), and 364 DEGs were specific differentially expressed in VI037601 post *Xcv* inoculation at different time points (Fig 2A and S5 Table). Overall, the resistant genotype, VI037601, had greater number of DEGs at 6 hpi, especially up regulated DEGs whereas the susceptible genotype ECW had greater number of DEGs at 24 hpi (Fig 2A and 2B) suggesting that the host plant response to *Xcv* infection is different between the two genotypes. Moreover, these DEGs might contain the disease resistance gene(s), such as *Bs1*, which conferred resistance to *Xcv* in VI037601.

### Functional enrichment analysis of DEGs

**Fig 3:**
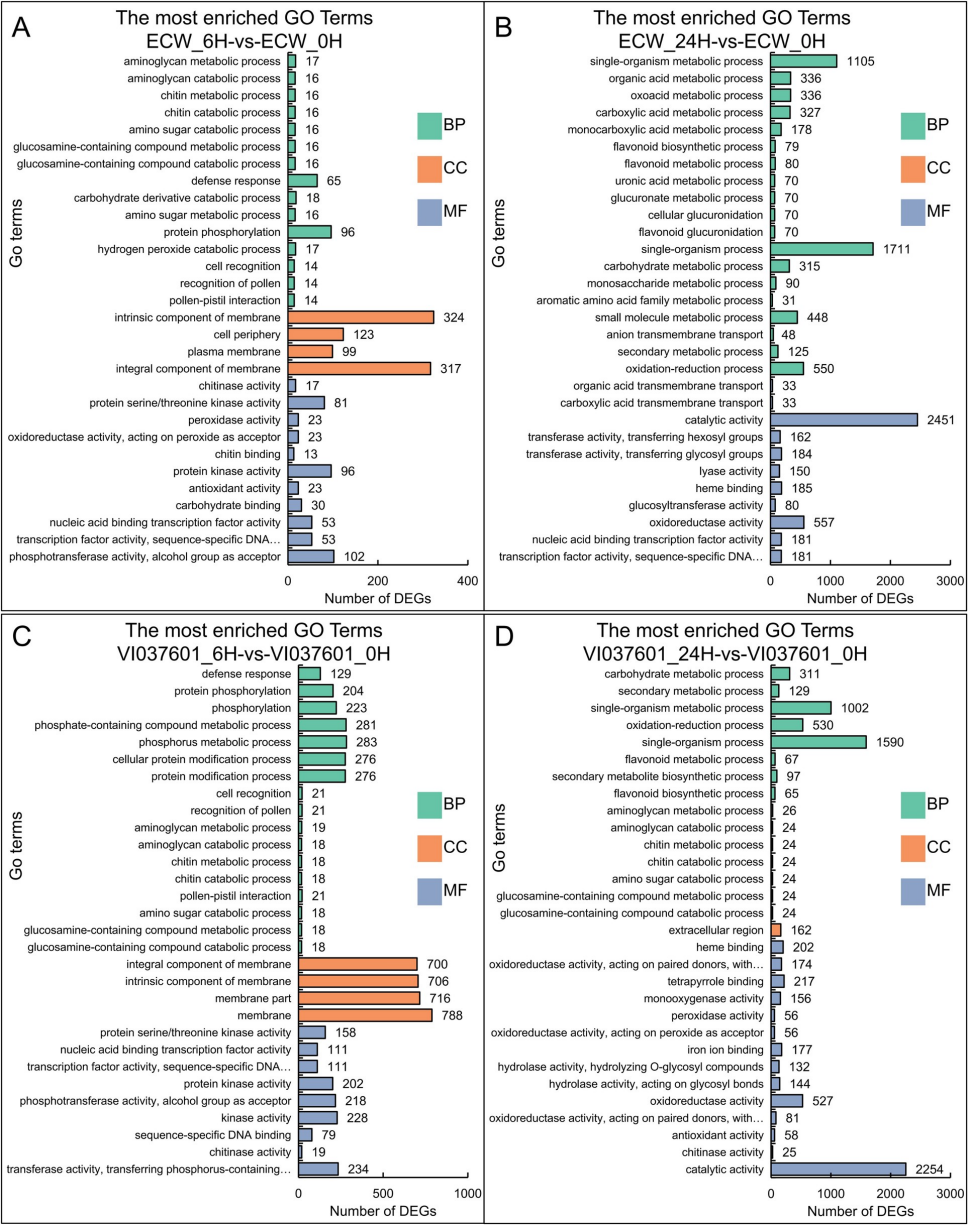
GO enrichment of DEGs in ECW and VI037601 post inoculation. GO classification of DEGs in group ECW_6H-vs-ECW_0H (**A**), group ECW_24H-vs-ECW_0H (**B**) and VI037601_6H-vs-VI037601_0H (**C**) and VI037601_24H-vs-VI037601_0H (**D**). The DEGs are summarized in three main categories: biological process (BP), cellular component (CC) and molecular function (MF). The X-axis indicates the number of genes and Y-axis indicates the GO terms.

In total, 6,334 of 11,232 DEGs in the four comparisons (ECW_6H-vs-ECW_0H, ECW_24H-vs-ECW_0H, VI037601_6H-vs-VI037601_0H and VI037601_24H-vs-VI037601_0H) were annotated with GO terms and assigned to three categorie (Fig 3 and S6 Table). The DEGs in VI037601_6H-vs-VI037601_0H were most enriched in defense response (GO:0006952), protein phosphorylation (GO:0006468), protein modification process (GO:0036211) in BP categories, integral component of membrane (GO:0016021) and intrinsic component of membrane (GO:0031224) in CC categories, protein kinase activity (GO:0004672), protein serine/threonine kinase activity (GO:0004674), and transcription factor activity, sequence-specific DNA binding (GO:0003700) in MF categories (Fig 3C and S6 Table). GO functional enrichment analysis of the ECW_6H-vs-ECW_0H group revealed a similar classification as the VI037601_6H-vs-VI037601_0H group (Fig 3A and S6 Table). The DEGs in VI037601_24H-vs-VI037601_0H were most enriched in single-organism process (GO:0044699), single-organism metabolic process (GO:0044710), carbohydrate metabolic process (GO:0005975), secondary metabolic process (GO:0019748) in BP categories (Fig 3B). Catalytic activity (GO:0003824) and oxidoreductase activity (GO:0016491) dominated MF categories (Fig 3D and S6 Table). The similar GO enrichment classification to VI037601_24H-vs-VI037601_0H groups were also found in ECW_24H-vs-ECW_0H (Fig 3B and S6 Table). These processes associated with disease resistance were enriched, indicating that the corresponding genes of these significant terms might play important roles in resistance to *Xcv* inoculation.

**Fig 4:**
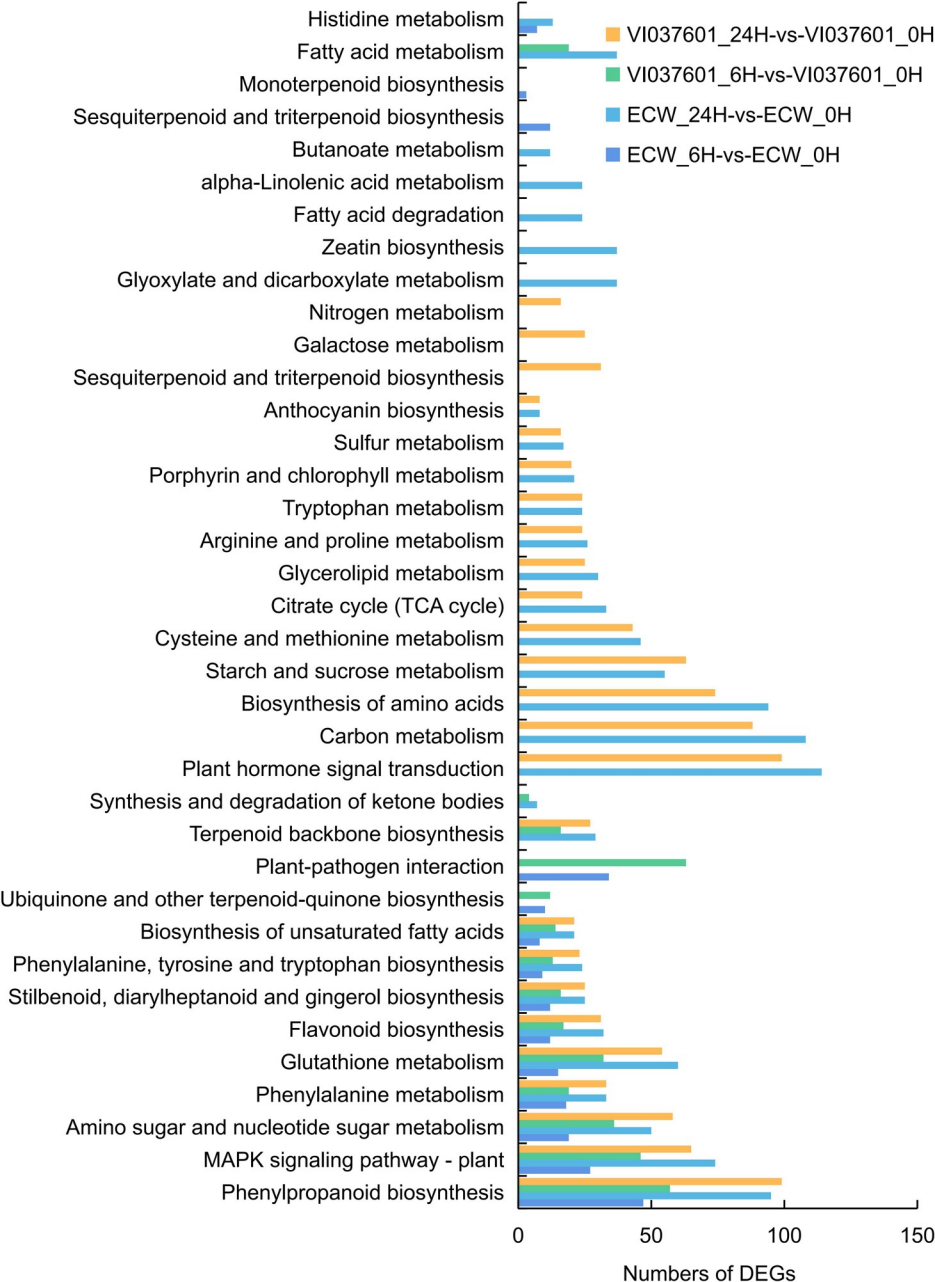
The significantly enriched KEGG pathway of DEGs in the four comparisons.

The significant KEGG enrichment pathways categories in the four comparisons were represented in this study. DEGs were significantly enriched in phenylalanine metabolism (ko00360), phenylalanine, tyrosine and tryptophan biosynthesis (ko00400), phenylpropanoid biosynthesis (ko00940), flavonoid biosynthesis (ko00941), stilbenoid, diarylheptanoid and gingerol biosynthesis (ko00945), glutathione metabolism (ko00480), biosynthesis of unsaturated fatty acids (ko01040) and MAPK signaling pathway (ko04016) in the four comparisons (Fig 4 and S7 Table). Plant-pathogen interaction (ko04626), ubiquinone and other terpenoid-quinone biosynthesis (ko00130), and monoterpenoid biosynthesis (ko00902) were enriched in ECW_6H-vs-ECW_0H and VI037601_6H-vs-VI037601_0H (Fig 4 and S7 Table). However, plant hormone signal transduction (ko04075), synthesis and degradation of ketone bodies (ko00072), and fatty acid metabolism (ko01212) were enriched in ECW_24H-vs-ECW_0H and VI037601_24H-vs-VI037601_0H (Fig 4 and S7 Table). Terpenoid backbone biosynthesis (ko00900), synthesis and degradation of ketone bodies (ko00072), and fatty acid metabolism (ko01212) were enriched in ECW_24H-vs-ECW_0H and VI037601_6H-vs-VI037601_0H. Moreover, many “Metabolism process” were also enriched in 24 hpi in ECW and VI0378601, such as carbon metabolism (ko01200), biosynthesis of amino acids (ko01230), starch and sucrose metabolism (ko00500), arginine and proline metabolism (ko00330), and porphyrin and chlorophyll metabolism (ko00860) (Fig 4 and S7 Table). These results indicated that the different expression patterns of DEGs in significant KEGG enrichment pathway categories in ECW and VI037601 helped to determine the functions of DEGs and screen of candidate resistance genes, which was responsible for the resistance to *Xcv* in VI037601.

### Transcriptional changes in response to Xcv infection

**Fig 5:**
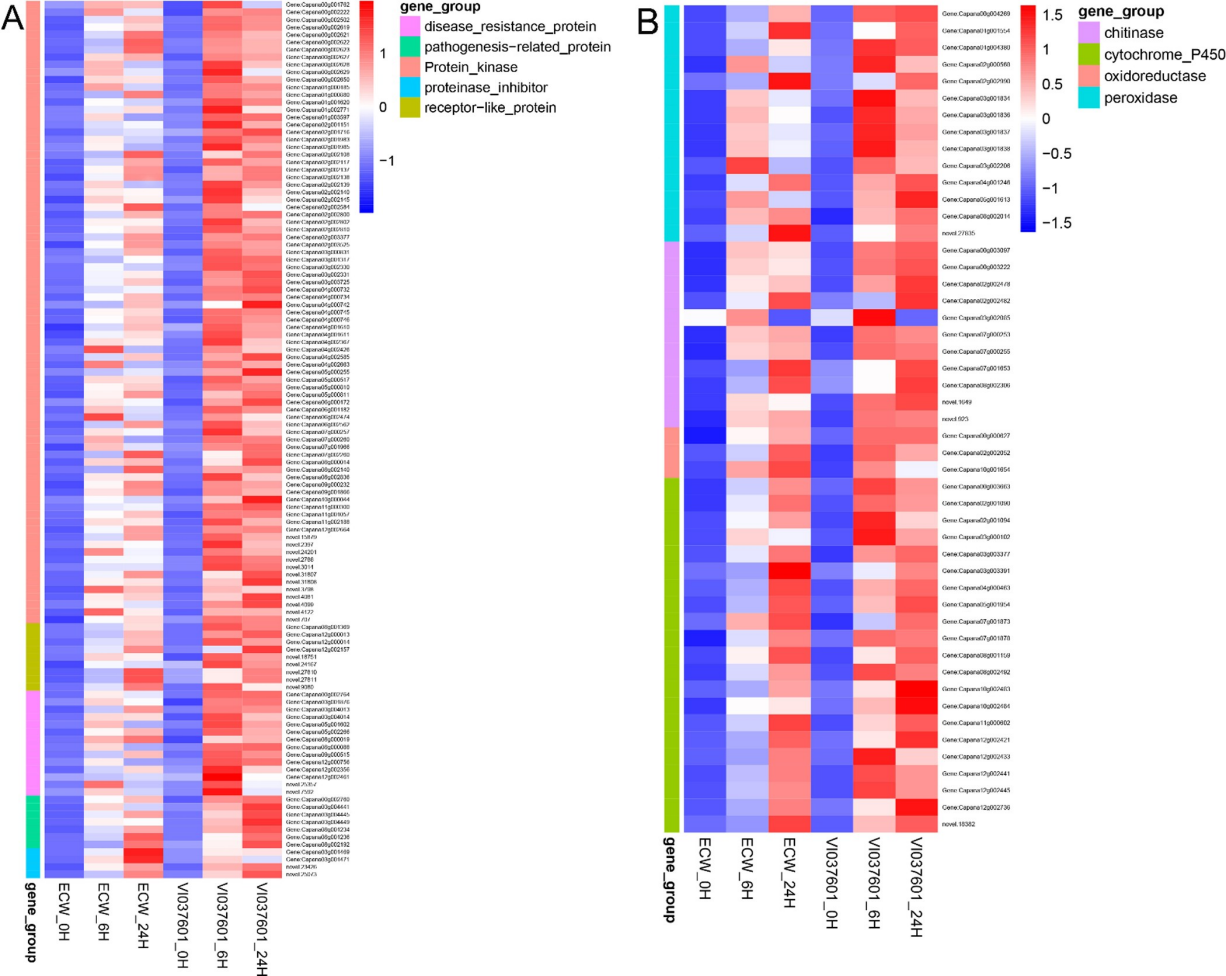
Heatmaps of the overlapping differentially expressed genes (DEGs) associated with disease resistance in ECW and VI037601 after *Xcv* inoculation. (**A**) DEGs encoding receptor like protein, protein kinase, disease resistance protein, proteinase inhibitor and pathogenesis-related protein. (**B**) DEGs encoding peroxidase, oxidoreductase and cytochrome P450. The color gradient represents the normalized FPKM value (Z-score) of DEGs (high expression (red) and low expression (blue)).

Many genes play a critical role in recognizing pathogen-associated molecular patterns (PAMPs) and subsequently activating plant defense mechanisms in response to pathogen attacks, such as kinases, pathogenesis-related (PR) protein, oxidoreductase and E3 ubiquitin-protein ligase [23, 40]. In this study, 541 pattern recognition receptors (PRRs), 30 MAPK, 246 resistance proteins (R Proteins), and 83 calcium signaling genes were identified by searching the keywords in the gene annotation (S8 Table). Among these DEGs, 24 protein kinases, 13 disease resistance proteins and 4 receptor-like proteins were specific differentially expressed in VI037601 post *Xcv* inoculation (S9 Table). We also found that Capana00g000272 (calcineurin B-like protein), Capana04g001405 (carboxylesterase), Capana09g000319 (aldehyde dehydrogenase), and Capana09g000326 (glycosyltransferase) were significantly differentially expressed in VI037601 post *Xcv* inoculation, but almost not expressed in ECW (S5 Table). Moreover, 83 overlapping differentially expressed kinase response genes, including 13 LRR receptor-like ser/thr protein kinase, were identified and up regulated in ECW and VI037601 at 6 hpi and 24 hpi (Fig 5A and S9 Table). Besides that, 30 common DEGs encoding other disease response proteins were also identified in ECW and VI037601 at 6 hpi and 24 hpi, such as disease resistance proteins, pathogenesis-related proteins and receptor-like proteins (Fig 5A and S9 Table). Interestingly, all of these DEGs were up-regulated at different time points after *Xcv* inoculation in ECW and VI037601 (Fig 5A and S9 Table). Other overlapping BS disease response genes, including 21 DEGs encoding cytochrome P450, 9 DEGs encoding E3 ubiquitin-protein ligase, 3 DEGs encoding oxidoreductase and 11 DEGs encoding chitinase were also differentially expressed and their expression levels increased after *Xcv* infection in ECW and VI037601 (Fig 4B and S9 Table). However, the expression analysis of *Bs* resistance genes showed that *bs2* (Capana09g000438) and *bs3* (Capana02g001306) were not/hardly expressed in pepper leaves before and after *Xcv* inoculation. Furthermore, the expression of their homologs did not change significantly (S3 Table).

### The response of differentially expressed transcription factors to Xcv infection

**Fig 6:**
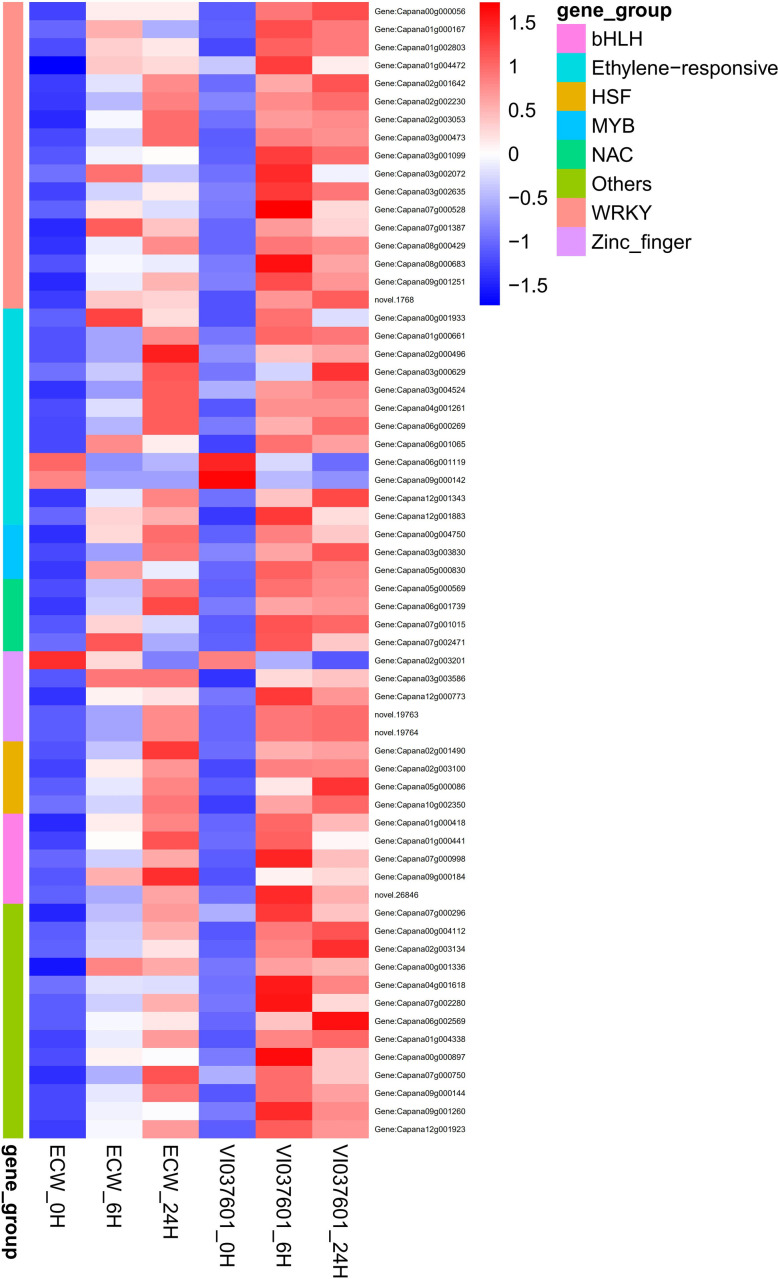
Heatmaps of the overlapping differentially expressed transcription factor genes (DEGs) in ECW and VI037601 post *Xcv* inoculation. The color gradient represents the normalized FPKM value (Z-score) of DEGs (high expression (red) and low expression (blue)).

In plants, transcription factors (TFs) play important roles in the regulation of different physiological and biochemical programs in response to plant-pathogen interaction [23]. In our study, 551 DEGs involving 53 TF families were identified, which included 69 zinc finger proteins (ZFPs), 65 ethylene-responsive transcription factor (ERFs), 64 MYB, 32 NAC, and 46 WRKY TFs (S10 and S12 Tables). Among them, most DEGs were up regulated in the four groups (S10 Table). Moreover, 21 TFs, including ZFPs, MYB, WRKY and ERFs were specific differentially expressed in VI037601 post *Xcv* inoculation (S9 Table). These identified TFs might be likely to perform an important role in pepper -*Xcv* interaction. Besides that, 63 putative overlapping TFs were differentially expressed in ECW and VI037601 after *Xcv* infection (Fig 6 and S9 Table). Interestingly, among these differentially expressed TFs, all DEGs encoding MYB, WRKY, ethylene responsive factor (ERFs), HSF, MYB, and bHLH TFs were up regulated after *Xcv* infection in ECW and VI037601, except for Capana06g001119 (ERF), Capana09g000142 (ERF), and Capana02g003201(ZFPs) (Fig 6 and S9 Table). Thus, enhanced activity of these up-regulated TFs suggests that they may be regulated in multiple ways by cis-acting sequences in response to *Xcv* infection. Nevertheless, different down regulated DEGs might also play an important role by negatively regulating the pepper immunity upon *Xcv* infection in ECW and VI037601.

### Validation of RNA-seq data by qRT-PCR

**Fig 7:**
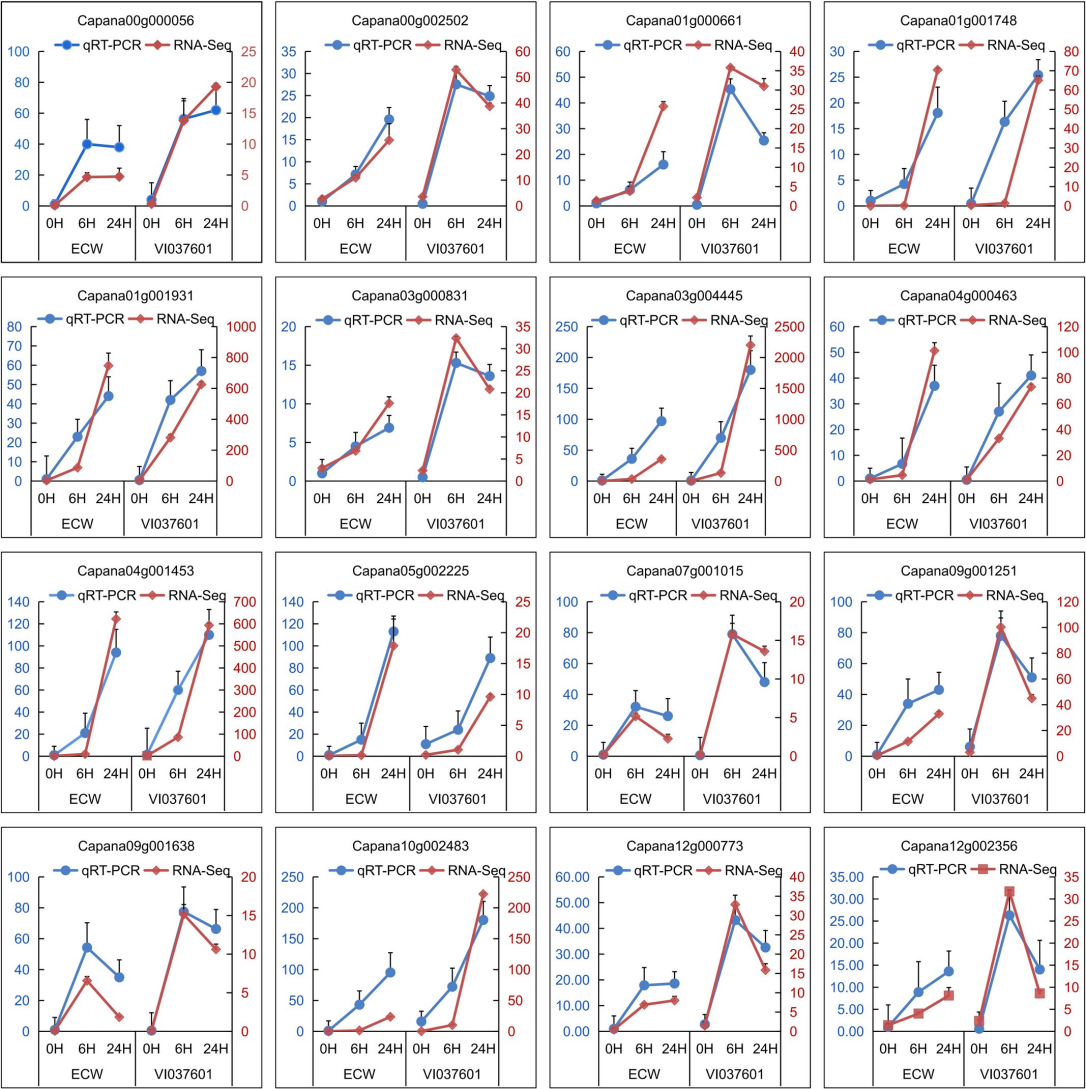
qRT-PCR based validation of DEGs in response to *Xcv* inoculation at different time intervals. Right ordinate (in red) represents the FPKM value of RNA-Seq. Left ordinate (in blue) represents the relative expression level of qRT-PCR. The expression level of genes in ECW at 0 hpi was set as 1.0, and other samples were calculated accordingly. The abscissa represents 0 hour, 6 hours and 24 hours (from left to right) post *Xcv* inoculation in ECW and VI037601. Data were represented as mean ± SD for three biological replicates.

To confirm the accuracy of RNA-seq data, transcriptional levels of 16 randomly selected DEGs representing a wide range of expression levels and patterns were detected in ECW and VI037601 post *Xcv* inoculation by qRT-PCR analysis (Fig 7). Among these 16 selected genes, majority of these DEGs were associated with massive defense response processes including receptor kinase (Capana01g001931 and Capana09g001638), protein kinase (Capana00g002502 and Capana03g000831), pathogenesis-related genes (Capana03g004445 and Capana04g001453), ERF (Capana01g000661), MYB TF (Capana05g002225), NAC TF (Capana07g001015), WRKY TF (Capana09g001251 and Capana00g000056), zinc finger protein transcription factor (Capana12g000773), disease resistance protein (Capana12g002356), and secondary metabolite biosynthesis (Capana01g001748, Capana04g000463 and Capana10g002483) (Fig 7). The fold changes varied in RNA-Seq and qPCR analyses. However, the expression data provided by qRT-PCR were following the profiles detected by RNA-seq at all time points in ECW and VI037601. These results suggested the reliability of RNA-seq to analyze the transcriptome of resistant and susceptible plants after *Xcv* infection.

## Discussion

Plants are exposed to a myriad of pathogenic microorganisms during their lifespan, including bacteria, fungi, viruses and nematodes, all of which try to acquire nutrients from the host plant for their advantage [41]. BS caused by *Xcv* is a very serious global disease, which has caused enormous yield and economic losses in pepper production, especially in regions with a warm and humid climate. In response to bacterial attack, plants deployed various defense responses, which were mainly activated by two branches of their immune system. One was the transmembrane pattern recognition receptors (PRRs) that initiated immune responses upon recognition of extracellular pathogen-associated molecular patterns (PAMPs). It was common to many kinds of microbes. The other was the products of resistance (R) genes that specifically recognized corresponding pathogen effectors, which were regarded as avirulence (Avr) factors [42]. Previous study revealed that cultivar carried *Bs1* was considered as a resistant material to BS due to hypersensitive response reaction to *Xcv* containing *avrBs1*, while non-hypersensitive reaction in response to infiltration of the bacterial suspension into leaf tissues appeared in ECW [43], which was consistent with the results in this study (Fig 1). However, the molecular mechanism of VI037601 and ECW in response to *Xcv* infection was unclear. In this study, RNA-seq technique was used to identify the DEGs associated with disease response during *Xcv* infection in the leaves of ECW and VI037601. The Q30 of clean reads in 18 samples were at least 89.3% and the mapping ratio of samples to the reference genome was above 91% (S1 Table), indicating that the quality of sequencing data was reliable. Here, the average products of each sample was 6.9 Gb exceeding the sample with 4.9 Gb successfully used to gain insight into CMV infection genes [23], indicating that the sequencing depth was sufficient for the transcriptome coverage. Although the fold change of the gene in qRT-PCR analysis was inconsistent with that in RNA-Seq. The trend of change was consistent in qRT-PCR and RNA-Seq, which indicated that the RNA-seq data was reliable for analyzing the transcriptome of resistant and susceptible plants after *Xcv* inoculation (Fig 7). Therefore, our transcriptome data is competent to analyze the defense-related genes and pathways against to *Xcv* in pepper.

Plants have an innate immunity system to defend themselves against pathogens by a number of mechanisms, such as hypersensitive response (HR), induction of genes encoding PR and/or induced biosynthesis of secondary metabolites [44, 45]. The functional analysis of DEGs in ECW and VI037601 post *Xcv* inoculation demonstrated that many biological processes were influenced by pathogen infection (Fig 4 and S7 Table). Many disease resistance pathways were most enriched at 6 hpi in both ECW and VI037601, such as defense response (GO:0006952), protein phosphorylation (GO:0006468), protein modification process (GO:0036211), protein kinase activity (GO:0004672), protein serine/threonine kinase activity (GO:0004674), and transcription factor activity, sequence-specific DNA binding (GO:0003700). Moreover, many secondary metabolic pathways such as oxidoreductase activity (GO:0016684), acting on peroxide as acceptor (GO:0016684), peroxidase activity (GO:0004601), chitinase activity (GO:0004568), chitin binding (GO:0008061), chitin catabolic process (GO:0006032), and chitin metabolic process (GO:0006030) were enriched at 24 hpi both in ECW and VI037601 (Fig 3 and S6 Table). Similar results were obtained in the previous study of tomato in response to infection by *Xanthomonas* perforans Race T3 [27]. The expression of these defense response genes induced the synthesis of secondary metabolites, which could inhibit the spread of *Xcv* in peppers. However, the number of enriched DEGs in VI037601 were more than twice the number of enriched DEGs in ECW in many significantly enriched GO terms, such as defense response, protein phosphorylation, protein modification process, protein serine/threonine kinase activity and transcription factor activity, sequence-specific DNA binding (Fig 3 and S6 Table). These DEGs might conferred the resistance of VI037601 to *Xcv*.

In plants, HR is a form of programmed cell death (PCD) at the site of pathogen infection, which is closely related to active resistance [46]. Previous studies showed that *Bs2* and *Bs3* were only expressed in BS resistant pepper post *Xcv* inoculation, which could trigger HR [16, 17]. Here, transcriptome profiling analysis results showed that *bs2* (Capana09g000438) and *bs3* (Capana02g001306) were not/hardly expressed in pepper leaves before and after *Xcv* inoculation (S3 Table). Moreover, the expression of their homologs also did not change significantly (S3 Table). The results indicated that *Bs1* gene that conferred resistance to *Xcv* in VI037601 may not be a homolog of *Bs2* and *Bs3*. However, many proteins kinases/enzymes encoded by DEGs were involved in defense-related gene induction and innate immunity, such as those that activate genes coding for the receptor-like kinases (RLKs), NAC TFs, WRKY TFs, pathogenesis-related protein and chitinase, as reported previously [23, 47–49]. In this study, 1,599 potentially defense-related genes linked to pattern recognition receptors (PRRs), mitogen-activated protein kinase (MAPK), calcium signaling, and transcription factors may regulate pepper resistance to *Xcv*. Moreover, 364 DEGs including protein kinase, oxidordeuctase, TFs and uncharacterized proteins were specific differentially expressed in VI037601 post *Xcv* inoculation, such as Capana02g003523 (receptor-like protein kinase), Capana02g000918 (WRKY), Capana12g000410 (peroxidase) and Capana01g000533 (uncharacterized protein) (S5 and S9 Tables). Interestingly, Capana00g000272 (calcineurin B-like protein), Capana04g001405 (carboxylesterase), Capana09g000319 (aldehyde dehydrogenase) and Capana09g000326 (glycosyltransferase) were also specifically expressed in VI037601, and the expression of which was significantly up-regulated after *Xcv* inoculation (S5 Table), indicating that they might play an important role in response to *Xcv* infection in VI037601. Receptor-like kinases are key pattern recognition receptors in response to pathogens [50]. Our findings also showed that many receptor-like kinases were significantly differentially expressed in ECW and VI037601, such as G-type lectin S-receptor-like serine/threonine-protein kinase (Capana07g002260) and LRR receptor-like serine/threonine-protein kinase (Capana03g000831), which were up regulated in ECW and VI037601 post *Xcv* inoculation (Fig 4 and S5 and S8 Tables). RLKs were important signaling components that played key roles in adapting to numerous biotic and abiotic stresses as well as in regulating plant growth and development [42, 51]. Generally, the mitogen-activated protein kinase (MAPK) cascades were initiated by the stimulated receptors. After a series of cascades reactions, activated MAPKs phosphorylated their substrates, most of which were enzymes and transcription factors, thereby triggering downstream responses [52].

WRKY TFs as the substrates of MAPKs can be regulated by MAPKs at transcriptional and/or post-translational levels [53–55]. For instance, OsWRKY53 was activated by OsMPK3 and OsMPK6 through transcriptional induction and phosphorylation in the process of pathogen infection, thereby enhancing rice resistance to pathogens [52, 56]. Here, 17 WRKY TFs were also up-regulated in ECW and VI037601 post *Xcv* inoculation (Fig 5 and S5, S9 and S10 Tables), which might be induced by MAPKs. Up-regulated expression of these WRKY TFs could activate downstream disease response genes or hormones pathway-related genes to protect against pathogen infection [40]. Besides, many studies showed that TFs that contain the NAC domain played pivotal roles in the regulation of the transcriptional reprogramming associated with plant stress responses, such as abiotic stress response and pathogen defense [57]. These NAC proteins might positively regulate plant defense responses by activating PR genes. One such example is ATAF1, which positively regulated penetration resistance to biotrophic fungus *Blumeria graminis* f.sp. *hordei* (*Bfh*) [58]. *OsNAC6*, *ONAC066*, *ONAC122*, *ONAC131*, and *OsNAC4* have been validated to be involved in defense responses against pathogen attack [59–62]. Here, the homologs of these NAC TFs were upregulated post *Xcv* inoculation in ECW and VI037601, such as Capana06g001739, Capana05g000569 and Capana11g001813 (S9 and S10 Tables). MYB TFs also played important roles in response to pathogen infection. Overexpression of *SmMYB44* in eggplant increased the resistance to bacterial wilt [63]. Previous study showed that TFs from the stress-related families ERFs, bZip, MYB and WRKY closely associated with the non-host response to *Xanthomonas campestris* pv. *vesicatoria* in *Citrus sinensi* [48]. Here, two MYB TFs were differentially expressed in VI037601, while three MYB TFs were up-regulated in both VI037601 and ECW post *Xcv* inoculation (Fig 6 and S9 and S10 Tables), which might play pivotal roles in non-host response to *Xcv*.

Moreover, phytohormones serve as key factors in plant responsiveness to stresses, and hormone-responsive genes are often used to qualitatively and quantitatively evaluate disease resistance responses during pathogen infection [64–68]. Plant resistance to biotrophic pathogens is positively regulated by ethylene and is negatively regulated by the auxin signal transduction pathway [64, 65]. Ethylene-responsive transcription factors mediated disease resistance was demonstrated in Arabidopsis and tomato against *Botrytis cinerea* and *Ralstonia solanacearum*, respectively [66–68]. Phytohormones were also involved in the non-host response of *Citrus sinensis* to *Xanthomonas campestris* pv. *vesicatoria* [69]. Here, 112 DEGs involved in plant hormone signal transduction were identified (S8 Table), and the results showed that almost DEGs involved in ABA, ETH, GA, and SA signal transduction were up regulated, while most DEGs participated to IAA and CTK signal transduction were down regulated after *Xcv* inoculation (S8 Table). Thus, these DEGs might implicate their roles in the regulation of transcriptional reprogramming associated with the response to *Xcv* in pepper.

## Conclusions

In this study, we performed a transcriptome analysis to reveal the defense related genes and pathways of resistant and susceptible pepper varieties after *Xcv* inoculation. A total of 120.23 Gb clean bases were generated and 11,232 DEGs were identified in 18 libraries. DEGs involved in PRRs, MAPK signaling, calcium signaling, phytohormone signaling pathways, TFs pathways and secondary metabolism, which were reported previously as relevant to defense response, were explored. To our best knowledge, this is the first study that examined global transcriptional changes in pepper plants infected with *Xcv*, which provides new knowledge and ideas for improving of peppers to avoid BS.

## Supporting information

S1 Table Statistic analysis of pepper clean reads in 18 libraries for RNA-seq. (XLSX)

S2 Table Detailed list of novel genes. (XLSX)

S3 Table Genes FPKM value, annotation, and function enrichment. (XLSX)

S4 Table Detailed list of DEGs in ECW and VI037601 at 6 hpi and 24 hpi relative to 0 hpi. (XLSX)

S5 Table Detailed list of specific differentially expressed genes in VI037601 post *Xcv* inoculation and overlapping DEGs in ECW and VI037601 at 6 hpi and 24 hpi relative to 0 hpi. (XLSX)

S6 Table Summary of GO terms related to the differentially expressed genes in ECW and VI037601 post *Xcv* inoculation. (XLSX)

S7 Table Significantly enriched KEGG pathway in ECW and VI037601 after infection of *Xcv*. (XLSX)

S8 Table Expression analysis of pepper (*Capsicum annuum* L.) defense-related genes and hormone-related genes. (XLSX)

S9 Table DEGs related to BS disease resistance that specific differentially expressed in VI037601 or commonly differentially expressed in VI037601 and ECW. (XLSX)

S10 Table Differentially expressed transcription factors in VI037601 and ECW after *Xcv* inoculation. (XLSX)

S11 Table qRT-PCR primers for the validation of RNA-Seq data. (XLSX)

S12 Table The number of DEGs identified as transcription factors in pepper. (XLSX)
